# Artificial Intelligence for Discovery in Life Sciences

**DOI:** 10.1021/acs.bioconjchem.6c00230

**Published:** 2026-07-01

**Authors:** Sushovan Chanda, Silvio O. Rizzoli, Ali H. Shaib

**Affiliations:** † Department of Neuro- and Sensory Physiology, 27177 University Medical Center Göttingen, Göttingen 37073, Germany; ‡ Center for Biostructural Imaging of Neurodegeneration, University Medical Center Göttingen, Göttingen 37073, Germany

## Abstract

Artificial intelligence is becoming a transformative tool in life sciences, not just by improving the results of existing technologies but also by introducing fundamental new ways of discovery. Initially applied to denoising, segmentation, or pattern recognition, it now extends across microscopy, structural biology, protein engineering, experimental design, and hypothesis generation. In imaging, deep learning enhances fluorescence, cryo-EM, and expansion microscopy and increasingly links optical and non-optical modalities. Beyond imaging, AI accelerates fluorescent probe development, while large language models and multi-agent systems are beginning to synthesize literature, generate hypotheses, and guide experiments. We survey these developments across imaging and non-imaging domains, from microscopy and structural biology to molecular design, hypothesis generation, and autonomous experimentation. We discuss the convergence of AI with tools from chemistry to instrumentation and explain challenges in validation, interpretability, generalizability, and autonomy. We conclude that AI is beginning to connect measurement, design, and reasoning to accelerate biological discovery.

## Graphical abstract

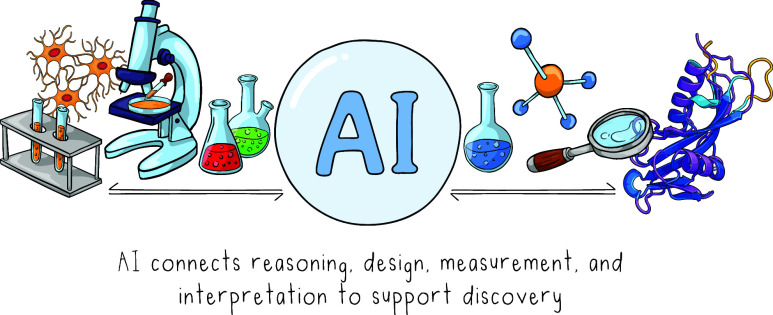

## Introduction

AI is no longer simply improving individual tasks in life sciences. It is beginning to reorganize how discoveries themselves are carried out. What started with denoising,^1,2^ segmentation,^3^ and pattern recognition^4^ has expanded into a much broader scientific role, reaching from microscopy and structural biology to molecular design, hypothesis generation, and experimental decision-making.^5–8^ This expansion matters not only because AI performs familiar tasks faster or at a larger scale but also because it increasingly links steps that were once separate: data acquisition, image interpretation, model generation, and experimental planning. In doing so, AI is shifting from a downstream analytical aid to an active component of the discovery process. Nowhere is this change more visible than in imaging, where AI no longer serves only to clean or segment data, but increasingly shapes reconstruction, multimodal integration, and the extraction of biological meaning,^8,9^ see Figure 1. At the same time, similar developments are unfolding well beyond microscopy, including probe development,^9^ protein engineering,^10^ literature synthesis,^11^ and autonomous research systems.^12^ This review examines these converging directions and argues that AI is not merely accelerating existing workflows but also helping to connect measurement, design, and reasoning within a more computationally supported discovery workflow.

**Figure 1:**
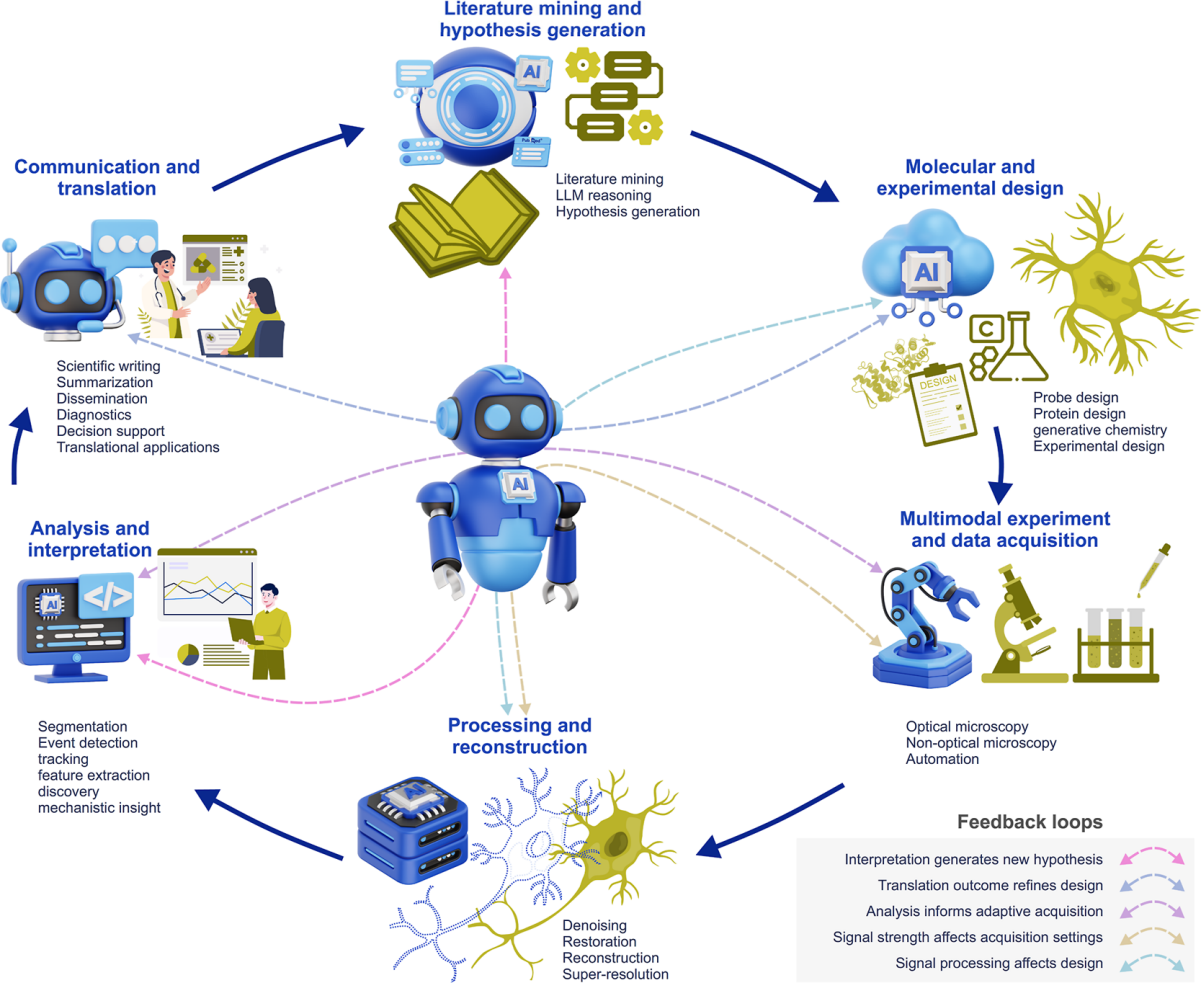
Emerging roles of AI across the life-science discovery workflow. Conceptual overview of areas in which AI methods are being applied or proposed across life-science research, from literature mining and hypothesis generation to experimental design, data acquisition, processing, interpretation, communication, and translation. The circular layout emphasizes potential feedback between downstream results and upstream decisions, without implying that these stages currently operate as a fully integrated autonomous pipeline.

## The AI-Powered Microscope: From Pixels to Insight

### Image Restoration and Denoising: From Content-Aware to Real-Time Deep Learning

One of the central constraints of fluorescence microscopy is the trade-off between the information content and photon burden. Improvements in spatial resolution, temporal resolution, or imaging speed frequently demand higher excitation intensities, increasing the likelihood of photobleaching and phototoxicity, especially in live-cell and in vivo imaging.^13^ As a result, many biologically relevant processes must be observed under conditions that are inherently suboptimal for image formation.^14^ Deep learning-based restoration has not removed this limitation, but it has substantially mitigated it by enabling the recovery of meaningful structural information from low-signal measurements.^15,16^

Content-aware image restoration (CARE)^15^ marked an early breakthrough by showing that deep networks could recover structurally informative images from photon-limited data. Trained on paired low-SNR and high-SNR images, CARE established low-dose imaging as a practical computational strategy and was subsequently extended across confocal^17^ and two-photon microscopy.^18^ The next advance was to remove the dependence on the clean reference data. Methods such as Noise2Noise and Noise2Void^19^ showed that denoising could be learned from corrupted or noisy images alone, greatly broadening their applicability in routine microscopy. More recently, denoising has moved from post-processing to direct integration within the imaging workflow. DeepCAD-RT^20^ exemplifies this shift by enabling real-time denoising during acquisition, thereby making restoration part of the measurement process. A further emerging direction is the use of diffusion probabilistic models, which extend restoration beyond that of conventional CNN-based denoisers. Although still at an early stage in biological imaging, these models point toward a more flexible and robust phase of generative reconstruction.^21^ Taken together, these developments show how AI-based denoising has progressed from offline post-processing to real-time, integrated hardware–software systems. This transition is particularly important for live-cell imaging, where biological dynamics must be captured under conditions that minimize light-induced damage (Table 1).

**Table 1:** AI Approaches for Recurring Image Analysis Challenges in Microscopy^a^

imaging problem	AI-based image solutions	references
low SNR and denoising	**supervised**: *CARE* (content-aware image restoration), a network trained on paired low/high-SNR images to restore signals. *DeepCAD-RT*, a real-time self-supervised 3D U-Net denoising framework that uses adjacent image frames for training and enables high-quality imaging under substantially reduced photon budgets. Self-supervised: *Noise2Void* trains on single noisy images to predict and cancel noise without a clean target. Deep learning denoisers significantly outperform classical filtering, achieving higher PSNR/SSIM and preserving true signal detail. **Denoising Diffusion Probabilistic Models**: *DDPM-SIM*, diffusion model trained on SIM raw images (3–5 frames), *CLASI*, two-stage diffusion (coarse and fine refinement) trained on cross-modality (widefield, TIRF, confocal) data. *Regularized diffusion*, trained with gradient-statistics regularization for filament fidelity. GAN + Contrastive **Learning**: Few-shot denoising using generative adversarial networks and structure-preserving loss functions. *SFSRM*, GAN with edge prior trained on paired diffraction-limited/STED images.	^15,19,20,22–28^
object detection	**specialized models**: *GFS-ExtremeNet,* a geometry-aware deep network that detects cells via extreme points, outperforming conventional detectors across varied cell shapes. *CryoFSL* preprint reporting few-shot SAM2-based cryo-EM particle picking with as few as five labeled micrographs. *CryoSIP*, semantic-instance collaborative network trained on cryo-EM micrographs with dual losses. **General detectors**: adapted versions of standard object detection CNNs (e.g., *SPHIRE-crYOLO* or *Faster R–CNN*) are also applied to microscopic images for tasks like cell or nucleus localization. **U** ^**2**^ **-Net** **with CBAM & Ghost Convolution**: lightweight and accurate salient object detection. **Uni-AIMS** **Platform**: robust detection of densely packed targets in cluttered microscopy environments.	^24,25,29–34^
image segmentation	U-Net **and variants**: The U-Net architecture revolutionized biomedical image segmentation, preserving fine details with its encoder and decoder design. Many extensions (3D U-Net, attention-U-Net, etc.) and instance segmentation models (Mask R-CNN, StarDist, Cellpose) have since emerged to handle complex microscopy data, dramatically improving cell and organelle segmentation accuracy. Modern approaches even incorporate transformers and foundation models for further gains. **SegNet and DeepLab**: popular architectures for semantic and instance segmentation. **DeepCut**: unsupervised segmentation using graph neural network clustering. **μSAM**: fine-tuned SAM on >17k bioimages (>2 M annotations). Cellpose-SAM: transformer backbone of SAM trained with Cellpose pipeline. STED-FM: ViT-MAE pretrained on 1M STED images, fine-tuned with 100 labels.	^35–47^
clustering and phenotyping	**unsupervised feature learning**: Deep learning enables clustering of images or pixels to discover phenotypes without predefined classes. For example, *CellCognition* Explorer uses a deep network to learn features from raw images and novelty detection to classify rare cell morphologies with no prior training. Similarly, Pixie is an unsupervised pipeline that clusters pixel-level features in multiplexed images to annotate tissue phenotypes automatically. These approaches reduce manual effort in grouping similar cells or patterns. **Optical Phenotyping via Deep Learning**: label-free classification using transcriptomic signatures. AI-Driven **Cell Feature Extraction**: enables clustering and phenotyping from spatial and morphological data.	^48–51^ https://software.cellcognition-project.org/
image reconstruction	**deep** **super-resolution** **and deconvolution**: CNN-based models and GANs have been trained to reconstruct high-quality microscopy images from diffraction-limited or noisy data, for instance, producing STORM-like super-resolved images from standard microscope inputs. **Physics-informed** **methods**: New hybrid approaches integrate optical physics into deep networks to improve fidelity. By incorporating PSF models or physical constraints into the loss, these methods can reduce the risk of hallucinated structures and improve reconstruction fidelity. **CryoDRGN &** **CryoDRGN-AI** **Tool**: deep generative models for continuous heterogeneity and ab initio reconstruction. **CryoFIRE Tool**: fast heterogeneous ab initio reconstruction using amortized inference. **SGM for PET**: a score-based model trained to reverse SDE conditioned on measurement data. **DeepEMhancer**: trained on pairs of raw cryo-EM maps and atomic-model-derived maps.	^52–59^

a Representative methods are grouped by imaging problem, including denoising, object detection, segmentation, phenotyping, and reconstruction. The examples illustrate how supervised, self-supervised, generative, foundation-model, and physics-informed models are being applied across microscopy and related structural imaging workflows.

### Pushing the Diffraction Limit: AI for Super-Resolution Microscopy

Deep learning has substantially expanded the scope of computational super-resolution microscopy.^8^ In many respects, this is a natural extension of the field, rather than a complete departure from it. Computational reconstruction has always been central to several forms of super-resolution microscopy, including structured illumination microscopy, where images are reconstructed from patterned illumination, and single-molecule localization microscopy, where molecular positions are inferred from sparse emitter signals.^60^ The same features that make AI useful for denoising and restoration are therefore also relevant to super-resolution, where the goal is to extract more information from limited, noisy, or sparsely sampled optical measurements. While hardware-based methods such as STED, dSTORM, and PALM can resolve structures at the lower double-digit nanometer scale, they often require specialized optics, high laser powers, or the acquisition of thousands of frames.^61^ AI-based approaches seek to recover high-frequency information directly from diffraction-limited data, reducing the acquisition burden and, in many cases, phototoxicity.

An early example was single-frame super-resolution microscopy,^23^ which showed that useful super-resolved information could be reconstructed from a single widefield image, making faster live-cell measurements more feasible than in localization-based approaches. More recent work has broadened this concept into more general and more controlled reconstruction frameworks. CLASI^27^ extends diffusion-based super-resolution across multiple imaging modalities, while regularized diffusion models^28^ address one of the field’s central problems, namely, the generation of visually convincing but structurally incorrect filamentous features. Similar developments are seen in structured illumination microscopy, where DDPM-SIM^26^ reduces the number of raw frames needed for reconstruction and Bayesian deep-learning SIM^62^ introduces pixel-level uncertainty estimates. AI is also beginning to influence STED microscopy, both through post-processing and through early efforts toward adaptive instrument control.^63,64^

More recently, the combination of expansion chemistry, optical fluorescence imaging, fluctuation analysis, and AI-based reconstruction has extended this logic from image enhancement toward molecular inference.^60,65^ In ONE microscopy,^66–68^ these components enabled three-dimensional reconstructions of individual protein molecules from light microscopy data, illustrating how AI-assisted optical microscopy can begin to approach structural biology when constrained by physical sample expansion and experimentally measured fluorescence signals.

Overall, these studies suggest that AI-based super-resolution is evolving from an isolated proof-of-concept enhancement into a broader class of reconstruction strategies. At the same time, the central risk is that AI may sharpen images beyond the information actually supported by the optical signal, fluorophore distribution, or labeling strategy. This is particularly important when apparent nanometer-scale precision is inferred from bulky labels, such as primary and secondary antibody complexes, whose effective linkage errors can be much larger than the claimed resolution. Robust validation against known geometries, simulated data, orthogonal measurements, and uncertainty estimates therefore remains essential because improved visual output does not necessarily guarantee structural correctness.^22^

### Foundation Models for Image Analysis: Segmentation, Detection, and Beyond

Classical segmentation approaches such as thresholding and watershed remain useful in well-controlled settings, but they often struggle with the heterogeneity of biological images across samples, cell types, and imaging modalities. Deep learning substantially improved this situation through models such as U-Net and its derivatives.^37^ More recently, foundation models have extended this shift by introducing large pretrained architectures that can be adapted to diverse segmentation tasks with relatively limited task-specific retraining. μSAM exemplifies this transition by adapting Meta’s Segment Anything Model^44^ for microscopy through separate generalist models for light and electron microscopy, integrated into a user-facing analysis framework. In parallel, Cellpose-SAM^45^ combines the Cellpose pipeline with an SAM-based transformer backbone, suggesting that transformer-based segmentation models can generalize well across varied imaging conditions, even if their performance still declines in especially crowded or low-contrast scenes. These approaches build on the Transformer architecture, which originated in sequence modeling^69^ and now underpins major developments in computer vision through Vision Transformers.^70^ The same architectural principles support foundation-model frameworks in biological image analysis, including SAM-derived models and ViT-based representation learning approaches. For super-resolution data, STED-FM^46^ extends the same principle to stimulated emission depletion microscopy, where self-supervised pretraining on a large STED image collection appears to reduce annotation demands for downstream tasks such as vesicle detection,^71^ mitochondrial segmentation,^72^ and dendritic spine classification.^73^ Overall, these developments suggest that foundation models are becoming an important component of microscopy image analysis. Although they do not eliminate the need for specialist architectures, they can reduce annotation requirements, broaden generalization across data sets, and lower the barrier to advanced segmentation workflows. Beyond supervised foundation models, unsupervised approaches such as *CellCognition* Explorer^48^ remain valuable for phenotype discovery, particularly when predefined classes are unavailable or when manual annotation is impractical. Representative imaging-focused advances are summarized in Table 2.

**Table 2:** Representative AI-Enabled Tools for Microscopy and Structural Imaging^a^

no.	tool	imaging domain	key advancement	references
1	content-aware image restoration (CARE)	fluorescence microscopy	a deep-learning-based denoising model that enables imaging with approximately 60-fold fewer photons while maintaining structural fidelity and enhancing both resolution and acquisition speed.	^15^
2	single-frame super-resolution (SFSRM) DL method	live-cell microscopy	a single-frame super-resolution method that produces resolution comparable to STED microscopy from diffraction-limited input data.	^23^
3	deep learning autofluorescence harmonic microscopy (DLAM)	label-free multiphoton microscopy	simultaneous multimodal imaging (2PA, SHG, 3PA) with improved spatial resolution and substantially reduced exposure time enabled by deep-learning-based enhancement.	^74^
4	SPHIRE-crYOLO	cryo-EM particle picking	automated fast particle detection in noisy cryo-EM micrographs using a YOLO-based architecture, achieving accuracy comparable to human annotation.	^31^
5	CryoFSL	cryo-EM particle picking	few-shot adaptation of SAM2 for cryo-EM segmentation, requiring only five labeled micrographs for effective fine-tuning. (Preprint)	^25^
6	CryoSIP	cryo-EM particle picking	a semantic-instance collaborative network trained with dual losses, reported to significantly reduce false-positive detections and improve particle-picking performance.	^29^
7	CryoSegNet (attention-gated U-Net + SAM)	cryo-EM particle picking	foundation-model-based segmentation that surpasses crYOLO and Topaz performance, enabling higher-quality 3D reconstructions with resolutions of approximately 3.32 Å.	^75^
8	REPIC consensus particle picking	cryo-EM	a graph-based consensus approach that integrates outputs from multiple particle pickers, improving selection reliability and enhancing final map quality.	^76^
9	AI image restoration for electron microscopy (CARE-EM)	electron microscopy	deep-learning restoration models trained on paired data sets markedly enhance EM image quality and improve downstream feature recognition.	^77^
10	ONE microscopy	expansion microscopy	reconstructing protein-level shapes from X10 expansion-microscopy data.	^66–68,78^
11	regularized diffusion	fluorescence SR	diffusion-based restoration model trained with gradient statistics regularization, yielding improved filament fidelity.	^28^
12	CLASI	fluorescence SR	cross-modality super-resolution at ∼100 nm achieved using a two-stage diffusion framework trained across widefield, TIRF, and confocal modalities.	^27^
13	DDPM-SIM	SIM	diffusion-based reconstruction from only 3–5 raw frames, trained on low-SNR SIM data sets to recover high-quality structured illumination images.	^151^
14	STED-FM	STED microscopy	preprint describing a ViT-MAE-based STED foundation model pretrained on nearly one million STED images and evaluated for label-efficient downstream tasks. (Preprint)	^46^
15	Cellpose-SAM	fluorescence microscopy	preprint reporting improved generalization relative to interhuman agreement across cellular segmentation benchmarks. (Preprint)	^45^
16	IVEA	live-cell fluorescence microscopy (TIRF/confocal)	automated detection of exocytosis events using a model pretrained on manually annotated data sets.	^79^
17	PROBY	fluorescence microscopy	graph-attention-based screening framework trained on combined protein–ligand and fluorophore data sets to support probe prioritization, candidate ranking, and targeted probe discovery.	^80^
18	denoising diffusion probabilistic models	fluorescence microscopy	diffusion models transform rough sketches into fully annotated synthetic microscopy images to train accurate AI segmentation models without manual annotation.	^81^
19	U^2^-Net	electron microscopy	an enhanced U^2^-Net variant incorporating attention and lightweight modules to automatically detect salient structures in microscopy images with high accuracy and reduced model size.	^26^
20	mask R-CNN	fluorescence microscopy	A mask R-CNN-based framework for simultaneous cell detection and segmentation, performing multiple end-to-end tasks through joint optimization of generic detection and instance-segmentation losses.	^82^

a The table summarizes selected AI-based methods across fluorescence microscopy, expansion microscopy, electron microscopy, cryo-EM, and label-free imaging. Tools are grouped by the imaging domain and highlight reported advances in denoising, super-resolution, particle picking, segmentation, reconstruction, event detection, and probe design.

### Intelligent Event Detection and Segmentation: From Static to Dynamic

A major challenge in biological imaging is that many objects of interest are dynamic, rather than static. Vesicle fusion, calcium transients, cell migration, and cell division can be difficult to annotate consistently and are often too numerous for manual analysis at a meaningful scale. AI-based tools are therefore increasingly used not only for structural segmentation but also for event detection in time-resolved data sets. IVEA^79^ provides one example of this shift, using pretrained deep-learning models to detect and classify exocytosis events in live-cell TIRF and confocal imaging while substantially reducing analysis time. More broadly, such systems illustrate how event-detection models can convert labor-intensive dynamic imaging assays into more tractable quantitative workflows. Scalability is also becoming increasingly important as biological image data sets grow from individual fields of view to tissues and large volumetric data sets. In this setting, graph-based learning strategies such as Cluster-GCN,^83^ which partition large graphs into manageable subgraphs for efficient training, point to ways of handling image-derived graphs at scales that would otherwise be computationally prohibitive. This is particularly relevant for connectomics and other high-volume imaging applications. Overall, these developments suggest that AI-based image analysis is extending beyond static segmentation toward the automated interpretation of dynamic and densely structured biological data. Although such approaches do not remove the need for validation, they substantially expand the scale and complexity of imaging experiments that can be analyzed in practice. The influence of AI across the microscopy pipeline is summarized in Table 3.

**Table 3:** AI Contributions across the Microscopy Workflow^a^

workflow stage	AI contributions (with examples)	references
sample preparation and probes	AI-designed **probes:** Machine learning is accelerating the design of fluorescent dyes and labels. For instance, the *FLAME* framework uses ML prediction models and generative chemistry to optimize fluorophore molecules, leading to new dyes with improved brightness and properties. By learning structure and property relationships, AI suggests chemical modifications or new compounds (some experimentally validated) that outperform traditional trial-and-error probe development. *PROBY*, graph attention network trained on protein–ligand docking + fluorophore properties. **Interpretable AI**: SHAP/attention maps trained on TICT/PICT fluorophores to reveal design rules.	^80,84,85^
imaging hardware and physics	**adaptive instrumentation**: AI techniques enhance the imaging process itself. One example is **adaptive optics (AO)** driven by neural networks; a custom ML controller can rapidly correct optical aberrations in microscopes in a sensor-less manner. In a recent approach, a physics-informed neural network was embedded in the microscope’s control loop, enabling real-time aberration correction across different imaging modalities. This *ML-AO* method outperformed conventional AO and proved robust even with 3D samples, sample motion, and low signal conditions. More generally, AI can optimize microscope settings (focus, illumination, etc.) and even suggest imaging protocols to maximize data quality. **Self-driving** **microscopes**: RL agents trained to adjust focus/illumination in real time.	^86,87^
image analysis and interpretation	**automated segmentation and analysis**: AI has transformed post-acquisition image analysis. Deep learning algorithms now automatically segment cells, nuclei, and other structures far more accurately and quickly than manual or classical methods. Tasks like feature extraction, object tracking, and classification can be handled by trained models, enabling high-throughput analysis of complex data sets. In fact, AI-driven analysis tools are becoming integral to microscopy workflows, improving both precision and efficiency from initial image processing to high-level quantitative insights. For example, generalist models (like the Segment Anything Model fine-tuned for microscopy) can adapt to diverse data, and AI-based analytics can reveal subtle phenotypic patterns that humans might miss. *Cellpose-SAM*, STED-FM, and *CryoFSL/CryoSIP*; see Table 1	^25,29,44–46,86,87^

a Representative examples show how AI is being applied at different workflow stages, including probe design, adaptive instrument control, image acquisition, segmentation, feature extraction, classification, and data interpretation.

## AI-Designed Matter: Probes, Labels, and Proteins

### Designing Fluorescent Probes: From Databases to Generative Chemistry

Fluorophore development has traditionally relied on iterative synthesis and testing across a large and chemically diverse design space.^88^ Because properties such as excitation and emission wavelengths, brightness, photostability, target specificity, and cell permeability must often be optimized simultaneously, probe design is inherently multidimensional.^89^ AI is increasingly useful in this setting because it can prioritize candidates, predict optical properties, and guide chemical exploration more efficiently than trial-and-error screening alone. FLAME^84^ illustrates this shift by combining a large fluorophore-solvent database with predictive and generative models for virtual screening, molecular generation, and structural optimization while also incorporating synthetic accessibility constraints to favor experimentally tractable candidates. PROBY^80^ extends this idea further by moving beyond fluorophore optimization alone and toward targeted probe design, jointly considering both optical performance and target interactions. This is an important conceptual step, although such frameworks still depend heavily on training data quality, docking realism, and downstream experimental validation.^80^ A persistent limitation in this area, however, is the limited interpretability of many machine-learning models, often referred to as the “black box” problem. In fluorophore design, one study^90^ addresses this using interpretable AI, including SHAP analysis and attention-based methods, to link model predictions to chemically meaningful structural features. Applied to TICT (twisted intramolecular charge transfer) and PICT (planar intramolecular charge transfer) fluorophores, this approach was used not only to rank candidates but also to extract design rules for subsequent fluorophore development. Together, these examples suggest that AI-assisted probe design is moving from candidate screening toward more target-aware and mechanistically informed chemical design. Beyond fluorescent probes, AI is also being applied to antibody–drug conjugate (ADC) development. Machine-learning models can support multiple stages of the ADC pipeline, including target identification, linker design, payload optimization, pharmacokinetic prediction, and clinical translation.^91,92^ These applications show how AI can assist the design of complex therapeutic systems in which molecular recognition, chemical stability, delivery, and biological efficacy must be considered together. Thus, AI-assisted molecular design is expanding beyond probe discovery toward broader applications in conjugate chemistry and targeted therapeutics.

### The Protein Design Revolution: From Structure Prediction to Generative Creation

Protein design has changed markedly with the rise of deep generative models. Rather than relying primarily on iterative mutation and screening,^93^ current approaches can generate candidate structures and sequences conditioned on desired geometries, motifs, or binding targets, substantially expanding the range of design problems that can be addressed computationally.^94–97^ A major step in this transition was RFdiffusion,^95^ which adapted structure-prediction networks into a generative denoising framework for protein backbone design. In doing so, it provided a flexible platform for tasks ranging from monomer design and symmetric oligomer assembly to enzyme active-site scaffolding and binder generation, helping establish generative modeling as a practical strategy for several benchmark protein-design tasks. RFdiffusion3 (RFD3),^94^ currently available as a preprint, extends this direction by modeling proteins in the context of ligands, nucleic acids, and other nonprotein partners. SeedProteo,^96^ reported as an arXiv preprint, explores all-atom generation and suggests that explicit side-chain and interface modeling may improve control over binder design. At the same time, targeted variants such as β-pairing RFdiffusion^97^ show that these models can also be steered toward specific unresolved challenges, in this case the design of binders to hydrophilic protein surfaces that are often poorly served by conventional interface-design strategies. Viewed together, these studies suggest that generative AI is not simply accelerating protein engineering, but reshaping its starting assumptions: from local sequence optimization toward model-guided exploration of structural and functional space. Even so, these advances do not make protein design routine or automatic, and their practical significance will continue to depend on careful experimental validation and on how well current benchmark successes generalize across more demanding biological contexts. In parallel, protein language models provide an important complementary strategy to structure-based generative design. Models such as ESM-2 ^98^ are trained on large protein sequence collections and learn relationships between sequence, structure, and function without requiring explicit structural supervision. They are increasingly used for function prediction, sequence optimization, and candidate prioritization, thereby broadening the computational toolkit for protein engineering.

## AI as a Scientific Collaborator: From Literature to Hypothesis

### Mining Literature and Generating Hypotheses: Beyond Keyword Search

The scale of the scientific literature now exceeds what any individual researcher can reliably track, especially across interdisciplinary fields. AI is therefore increasingly used not only to retrieve relevant papers but also to organize relationships across the literature, synthesize findings, and help formulate candidate hypotheses. This shift predates current LLM-based systems: early literature-based discovery efforts, beginning with Swanson’s work, showed that implicit links across separate literature studies could already be used to generate plausible biomedical hypotheses.^99^ Later systems such as DeepDive^100^ and Semantic Scholar^101^ built structured, queryable representations of entities and their relationships, enabling more semantic forms of literature search, while tools such as LitSense 2.0 extended retrieval beyond keyword matching toward concept-, sentence-, and passage-level search.^102^ These systems improved the organization and navigation of scientific knowledge, but their primary role remained supportive rather than generative. This distinction is important because it marks the transition to the next phase: once AI could structure and retrieve the literature at scale, attention turned to whether it could also help infer, prioritize, and formulate hypotheses from that knowledge.

Several recent systems begin to address this next step, although from different starting points. SKiM-GPT^103^ remains rooted in the literature itself, combining the co-occurrence structure with LLM-based evaluation to surface hypotheses that are implicit across many papers rather than stated in any one paper. Coated-LLM^104^ adds a more agentic layer, using multiple model roles to generate, challenge, and refine candidate ideas instead of accepting a single output at face value. Together, these approaches illustrate a cautious shift beyond search alone toward systems that can help structure uncertainty, prioritize candidate hypotheses, and support earlier stages of scientific reasoning.

### LLMs for Writing

LLMs are also beginning to shape scientific writing itself,^105–108^ but this is an area where caution is especially warranted. In constrained roles, such as language editing, structural refinement, abstract drafting, or citation-grounded literature synthesis, they can be genuinely useful, particularly when coupled to retrieval-based systems rather than free-text-generation alone. Retrieval-augmented scientific assistants such as OpenScholar^11^ illustrate the more defensible end of this spectrum by grounding synthesis in identified passages and citation-based responses. At the same time, fluent output should not be mistaken for the scientific value. In medical writing, ChatGPT-generated abstracts were found to be more readable yet inferior in overall quality to the originals.^109^ More broadly, the release of LLMs has been followed by a rapid rise in LLM-related medical publications,^110^ while large-scale analyses of more than 15 million biomedical abstracts suggest measurable shifts toward increasingly homogenized vocabulary in the post-LLM era:^105^ This may accelerate manuscript production, but it also adds strain to readers, reviewers, and editors by increasing the volume of publishable-looking text without necessarily increasing the level of interpretability or insight. The now-retracted Frontiers review on spermatogonial stem cells (10.3389/fcell.2023.1339390) provides an extreme but instructive example: concerns about the nature of its AI-generated figures led the journal to conclude that the article did not meet its standards of editorial and scientific rigor. This case illustrates that fluent or visually convincing AI-generated output can pass superficially as scholarly content while still failing to meet basic standards of scientific reliability.

### Multi-Agent Systems and Autonomous Discovery

AI-assisted hypothesis generation has also led to growing interest in multi-agent systems, in which specialized model roles propose, critique, filter, and refine candidate ideas. The appeal of these still-emerging architectures is that they make iteration, disagreement, and staged evaluation explicit parts of the computational workflow. AgenticSciML^111^ illustrates this direction in scientific machine learning benchmarks, while related agentic frameworks in molecular or materials settings couple language-model reasoning to simulation and filtering steps.^112^ SAGA^113^ extends this logic by allowing goal revision when intermediate results suggest that the original objective may be unproductive. Robin^114^ is presented as a lab-in-the-loop example linking literature search, hypothesis generation, experimental planning, and validation. This emerging literature is complemented by several established studies that have helped to define autonomous discovery as a broader field. Coscientist^115^ combines literature retrieval, planning, code execution, and experimental design within chemistry workflows, demonstrating how large language models can support multiple stages of laboratory-oriented scientific reasoning. ChemCrow^116^ similarly integrates large language models with chemistry-specific computational tools to assist in synthesis planning, molecular analysis, and scientific problem solving. A-Lab^117^ extends this direction into autonomous material discovery by coupling AI-guided planning with automated experimentation in a closed-loop setting. In a more computational setting, FunSearch^118^ shows how large language models can generate and refine solutions to mathematical and optimization problems through an iterative search process. AlphaEvolve,^119^ currently available as a preprint, further develops this program-search concept toward AI-guided optimization and refinement of candidate solutions. Together, these examples show how reasoning, computation, tool use, and experimentation are becoming increasingly integrated within autonomous discovery workflows. Considered together, these systems suggest that the main promise of multi-agent AI lies not in replacing scientific judgment but in structuring complex discovery tasks into iterative, semiautonomous workflows that are more scalable than manual reasoning alone, see Figure 1. Representative non-imaging examples discussed in these sections are summarized in Table 4.

**Table 4:** Emerging AI Approaches for Hypothesis Generation and Computational Discovery

no.	discovery/tool	imaging domain	key advancement	references
1	RFdiffusion3	protein design (structural biology)	RoseTTAFold model fine-tuned for protein–ligand denoising, enabling joint design with ligands and nucleic acids. (Preprint)	^94^
2	SeedProteo	protein design	all-atom diffusion framework trained on full atomic protein structures, with reported wet-lab hit rates of approximately 70–80% in two therapeutic-target case studies, PD-L1 and SC2RBD. (Preprint)	^96^
3	β-pairing RFdiffusion	protein design	design of binders for hydrophilic protein surfaces using a model fine-tuned with a β-pairing interaction bias.	^97^
4	SKiM-GPT	hypothesis generation	retrieval-augmented hypothesis-generation framework combining SKiM-based co-occurrence retrieval with GPT-4-based hypothesis evaluation and a Phi-3 mini relevance filter. Reported κ = 0.84 agreement in a benchmark involving 14 hypotheses evaluated by four raters.	^103^
5	Coated-LLM	drug combination discovery	multi-agent discovery. Three LLM agents fine-tuned on pharmacology literature.	^104^
6	statLens	hypothesis from omics data	technology report describing a closed-loop omics-guided refinement pipeline and reporting improved candidate prioritization in internal evaluations.	https://technologies.research.gwu.edu/technology/60709
7	HypoGen	hypothesis generation using structured paper data	HypoGen framework incorporating an explicit chain-of-reasoning module that formalizes the conceptual progression from “bit” to “flip”. (Preprint)	^120^
8	AgenticSciML	multi-agent scientific discovery	Scientific Machine Learning (SciML) combining data-driven inference with physics-based modeling to tackle complex scientific and engineering problems. (Preprint)	^111^
9	MIND	molecular discovery	review of Pareto-based algorithms for multiobjective molecular property optimization beyond drug discovery	^121^
10	SAGA	agent for scientific discovery	SAGA is presented as a bilevel agentic framework in which LLM agents derive and formalize candidate objectives, while an inner optimization loop searches for solutions under the updated goals. (Preprint)	^113^
11	Robin	multi-agent for scientific discovery	Robin describes a multi-agent, lab-in-the-loop framework for therapeutic discovery, in which AI-generated candidates are iteratively evaluated and refined through experimental validation. (Preprint)	^114^
12	SciLitLLM	literature understanding	SciLitLLM describes a continual pretraining and supervised fine-tuning framework for adapting LLMs to scientific literature understanding and task-specific instruction following. (Preprint)	^122^
13	Literature Meets Data	hypothesis generation	Literature Meets Data describes an LLM-based framework for combining literature-derived information with data-driven evidence to support the generation and prioritization of candidate hypotheses. (Preprint)	^123^
14	AI-assisted hypothesis generation	hypothesis generation in cardiotoxicity research	AI-assisted hypothesis generation for tackling cardiotoxicity challenges, demonstrated in a simulation study using GPT-4o.	^124^

## AI across Imaging Modalities

### Cryo-Electron Microscopy and Structural Biology

AI is now embedded across the cryo-EM workflow, from particle picking to post-processing and model building. Early gains came from particle picking, where methods such as SPHIRE-crYOLO^31^ and Topaz^125^ reduced one of the major manual bottlenecks in the pipeline. More recent systems such as CryoFSL^25^ and CryoSIP^29^ extend this by improving generalization, reducing false positives, and adding confidence-aware selection. After reconstruction, AI-based post-processing tools such as DeepEMhancer^57^ improve map interpretability, while model-building systems such as DeepTracer^126^ help convert density into structural hypotheses more efficiently.

A further step has been the integration of cryo-EM with protein structure prediction. AlphaFold,^127^ AlphaFold3,^128^ and related approaches are increasingly used to predict domains or subunits that can be docked into experimental density maps and refined further, particularly in multidomain proteins or partially resolved complexes.^129,130^ AlphaFold 3 further expands this integration by extending prediction beyond proteins alone to interactions involving ligands, nucleic acids, and other biomolecular partners. Together, these advances strengthen the link between predictive modeling and experimental structural biology. The effect of AI in cryo-EM has therefore been less a single breakthrough than a cumulative reorganization of the workflow, improving throughput, consistency, and interpretability across multiple stages. These gains, however, do not remove the need for careful validation, as model accuracy still depends strongly on map quality, particle heterogeneity, and the limits of the underlying experimental data.

### Connectomics and Volume Electron Microscopy

Connectomics places some of the heaviest demands on AI in imaging because reconstructing neural circuits requires the segmentation and tracing of densely packed processes across extremely large volumetric data sets. In electron microscopy, deep learning has become central to this workflow: methods such as Flood-Filling Networks^131^ and later 3D segmentation pipelines^132^ made large-scale neurite reconstruction practically tractable, supporting circuit mapping in data sets ranging from the fly brain^133^ to cubic-millimeter volumes of the mouse cortex.^134^ More recently, related AI-based strategies have also begun to support connectomic reconstruction from expanded light-microscopy data, pointing to possible links between circuit reconstruction and molecular information.^135^ Overall, AI has become essential in connectomics not only because of scale but also because reliable reconstruction across large volumes depends on automated tracing, segmentation, and error control.

### Atomic Force, Chemical, and Biomedical Imaging

AI is also extending into imaging systems beyond conventional optical microscopy. In atomic force microscopy (AFM),^136^ where images reflect surface topography and mechanical interactions at nanometer resolution, AI has been applied to adaptive scan control, denoising, classification, and property prediction.^137^ Reinforcement-learning strategies^138^ have been explored for real-time adjustment of scanning parameters, while deep networks such as U-Nets^139^ and transformers have been used to suppress scan noise and correct tip-related artifacts.^140^ Related machine-learning approaches^141^ have also been used to classify surface textures and infer mechanical or electrochemical properties directly from AFM data.^142,143^ In chemical imaging, including nanoSIMS, AI^142^ is increasingly used to address reconstruction and registration problems that arise when elemental or isotopic maps must be aligned with fluorescence data or extended into three dimensions. Ion beam tomography combined with deep-learning-based denoising and reconstruction has enabled high-resolution 3D chemical mapping of subcellular organization,^144^ illustrating how AI can help recover an interpretable structure from sparse or noisy acquisition regimes.^145^ In biomedical imaging, AI is used not only for reconstruction from noisy or incomplete measurements but also for the analysis of patient scans. In PET, score-based generative models,^146^ which derive from score-matching approaches for learning data distributions,^147^ operate in image reconstruction from partial acquisition data, yielding enhanced quality and quantitative fidelity relative to conventional iterative approaches. In parallel, machine-learning and deep-learning methods are increasingly being applied to MRI for lesion characterization and multiparametric image analysis,^148,149^ while breast-imaging workflows, including mammography, ultrasound, and MRI, are also being reshaped by AI-assisted detection and decision support.^150^ In many of these settings, however, evidence remains task-specific, and improvements in visual quality or downstream classification do not, by themselves, demonstrate improved physical accuracy. Overall, these examples show that AI is not tied to one imaging technology but to a recurring set of computational problems, including noise suppression, reconstruction from limited data, feature extraction, and inference from complex measurement spaces. Across scanning probe, chemical, and biomedical imaging, its role is increasingly to make difficult data more interpretable without changing the underlying physical constraints of the measurement.

## Conclusion

AI is becoming an increasingly important component of discovery in life sciences, spanning imaging, molecular design, hypothesis generation, and autonomous experimentation. As discussed throughout this review and summarized in Table 4, its influence now extends well beyond isolated analytical tasks and into the broader organization of research workflows. At the same time, the field faces substantial challenges in validation, interpretability, bias, generalizability, and the responsible use of increasingly autonomous systems. AI may also widen access to advanced research capabilities, particularly in settings where infrastructure or specialist support is limited, although this promise will depend on equitable access to reliable tools, data, and computing. Broader concerns also deserve attention, including the environmental costs of large-scale AI through energy and water consumption, as well as cognitive and epistemic risks if fluent outputs begin to displace rather than support critical scientific judgment. The long-term significance of AI in biology will therefore depend not only on technical progress but also on whether these systems are integrated with rigorous standards of evaluation, transparency, sustainability, and scientific oversight.

## References

[1] Vincent P., Bengio Y., Manzagol P.-A. (2008). Extracting and composing robust features
with denoising autoencoders. Proc. ICML.

[2] Vincent P., L H., Lajoie I., Bengio Y., Manzagol P.-A. (2010). Stacked denoising
autoencoders: Learning useful representations in a deep network with
a local denoising criterion. J. Mach. Learn.
Res..

[3] Piraino D. W., Amartur S. C., Richmond B. J., Schils J. P., Thome J. M., Weber P. B. (1991). Segmentation of magnetic resonance images using an
artificial neural network. Proc. Annu. Symp.
Comput. Appl. Med. Care.

[4] Fukushima K. (1980). Neocognitron:
a self organizing neural network model for a mechanism of pattern
recognition unaffected by shift in position. Biol. Cybern.

[5] Ashyrmamatov I., Gwak S. J., Jin S. Y., Jun I., Ucak U. V., Lee J. Y., Lee J. (2026). A survey on large language
models
in biology and chemistry. Exp. Mol. Med..

[6] Zhang Y., Khan S. A., Mahmud A., Yang H., Lavin A., Levin M., Frey J., Dunnmon J., Evans J., Bundy A., Dzeroski S., Tegner J. (2025). Exploring
the role of large language models in the scientific method: from hypothesis
to discovery. npj Artif. Intell..

[7] Wu X., Wu M. A., Zou J., Kleinstreuer N., Wu J. C. (2026). Reimagining human-centric drug development with new approach methodologies. Science.

[8] Li S., Meng X., Zhou B., Tian W., Chen L., Zhang Y. (2025). AI-empowered super-resolution
microscopy: a revolution in nanoscale
cellular imaging. Nat. Methods.

[9] Zhang S., Dai G., Huang T., Chen J. (2024). Multimodal large language models
for bioimage analysis. Nat. Methods.

[10] Singh N., Lane S., Yu T., Lu J., Ramos A., Cui H., Zhao H. (2025). A generalized platform
for artificial intelligence-powered
autonomous enzyme engineering. Nat. Commun..

[11] Asai A., He J., Shao R., Shi W., Singh A., Chang J. C., Lo K., Soldaini L., Feldman S., D’Arcy M. (2026). Synthesizing scientific
literature with retrieval-augmented language
models. Nature.

[12] Lu C., Lu C., Lange R. T., Yamada Y., Hu S., Foerster J., Ha D., Clune J. (2026). Towards end-to-end automation of AI research. Nature.

[13] Liu Z., Lavis L. D., Betzig E. (2015). Imaging live-cell
dynamics and structure
at the single-molecule level. Mol. Cell.

[14] Hoebe R. A., Van Oven C. H., Gadella T. W., Dhonukshe P. B., Van Noorden C. J., Manders E. M. (2007). Controlled light-exposure microscopy
reduces photobleaching and phototoxicity in fluorescence live-cell
imaging. Nat. Biotechnol..

[15] Weigert M., Schmidt U., Boothe T., Muller A., Dibrov A., Jain A., Wilhelm B., Schmidt D., Broaddus C., Culley S. (2018). Content-aware image restoration: pushing the limits
of fluorescence microscopy. Nat. Methods.

[16] Shroff H., Testa I., Jug F., Manley S. (2024). Live-cell imaging powered
by computation. Nat. Rev. Mol. Cell Biol..

[17] Laine R. F., Jacquemet G., Krull A. (2021). Imaging in focus: An introduction
to denoising bioimages in the era of deep learning. Int. J. Biochem. Cell Biol..

[18] Lu Y., Ying Y., Lin C., Wang Y., Jin J., Jiang X., Shuai J., Li X., Zhong J. (2025). UNet-Att:
a self-supervised denoising and recovery model for two-photon microscopic
image. Complex Intell. Syst..

[19] Krull, A. ; Buchholz, T.-O. ; Jug, F. Noise2Void - Learning Denoising From Single Noisy Images. 2019 IEEE/CVF Conference on Computer Vision and Pattern Recognition (CVPR), 2018; pp 2124–2132.

[20] Li X., Li Y., Zhou Y., Wu J., Zhao Z., Fan J., Deng F., Wu Z., Xiao G., He J. (2023). Real-time denoising
enables high-sensitivity fluorescence time-lapse
imaging beyond the shot-noise limit. Nat. Biotechnol..

[21] Guo Z., Liu J., Wang Y., Chen M., Wang D., Xu D., Cheng J. (2024). Diffusion
models in bioinformatics and computational biology. Nat. Rev. Bioeng..

[22] Wang H., Rivenson Y., Jin Y., Wei Z., Gao R., Gunaydin H., Bentolila L. A., Kural C., Ozcan A. (2019). Deep learning
enables cross-modality super-resolution in fluorescence microscopy. Nat. Methods.

[23] Chen R., Tang X., Zhao Y., Shen Z., Zhang M., Shen Y., Li T., Chung C. H. Y., Zhang L., Wang J. (2023). Single-frame deep-learning
super-resolution microscopy
for intracellular dynamics imaging. Nat. Commun..

[24] Jiang, H. ; Li, S. ; Liu, W. ; Zheng, H. ; Liu, J. ; Zhang, Y. Geometry-Aware Cell Detection with Deep Learning. mSystems 2020, 5 (1). DOI: 10.1128/msystems.00840-19. PMC7002118 32019836

[25] Poudel B., Gyawali R., Dhakal A., Cheng J., Xu D. (2025). CryoFSL: An
Annotation-Efficient, Few-Shot Learning Framework for Robust Protein
Particle Picking in Cryo-EM Micrographs. bioRxiv.

[26] Yan J., Tang Z., Yan Q., Huang S. (2026). Prior-enhanced diffusion
model for super-resolution reconstruction in structured illumination
microscopy. Opt Laser. Eng..

[27] Ren C., Pan T., Yang T., Yin N., Gu L., Liu B., Ji W. (2025). Combined learning for augmented super-resolution imaging. Opt. Laser Technol..

[28] Abgaryan M., Cui X., Gopan N., Della Maggiora G., Yakimovich A., Sbalzarini I. F. (2025). Regularized
Gradient Statistics Improve Generative
Deep Learning Models of Super Resolution Microscopy. Small Methods.

[29] Deng Y., Wang S., Xiang M., Li Y., Zhuo L., Cao D., Fu X., Zou Q. (2026). CryoSIP: unleashing protein high-resolution
Cryo-EM via semantic-instance collaborative picking. Briefings Bioinf..

[30] Liu M., Wang Y., Wang T. (2025). Ghost Convolutional Neural Network-Based
Lightweight Semantic Communications for Wireless Image Classification. IEEE Wireless Commun. Lett..

[31] Wagner T., Merino F., Stabrin M., Moriya T., Antoni C., Apelbaum A., Hagel P., Sitsel O., Raisch T., Prumbaum D. (2019). SPHIRE-crYOLO is a fast
and accurate fully
automated particle picker for cryo-EM. Commun.
Biol..

[32] Wen T., Wu H., Du Y., Huang C. (2020). Faster R-CNN with improved
anchor
box for cell recognition. Math Biosci Eng..

[33] Lin, Y. ; Segmentation algorithm for cancer regions in breast cancer MRI images based on the Improved U2-Net Network. Proceedings of the 2nd International Conference on Machine Learning and Automation, 2024.

[34] Stevens M., Nanou A., Terstappen L., Driemel C., Stoecklein N. H., Coumans F. A. W. (2022). StarDist Image
Segmentation Improves Circulating Tumor
Cell Detection. Cancers.

[35] He K. (2018). Mask R-CNN. ArXiv.

[36] Ozan O., Folgoc L. Le, Lee M., Heinrich M., Misawa K., Mori K., McDonagh S., Hammerla N. Y., Kainz B., Glocker B., Rueckert D. (2018). Attention
U-Net: Learning Where to
Look for the Pancreas. ArXiv.

[37] Ronneberger
Olaf F. P. (2015). Brox Thomas U-Net: Convolutional Networks for Biomedical
Image Segmentation. ArXiv.

[38] Yanhui
Hong N. W., Xia Z., Tao H., Fang Xi, Li Y., Wang J., Jin P., Cai X., Li S., Chen Z., Zhang Z., Ke G., Zhang L. (2025). Uni-AIMS:
AI-Powered Microscopy Image Analysis. ArXiv.

[39] Stringer C., Wang T., Michaelos M., Pachitariu M. (2021). Cellpose:
a generalist algorithm for cellular segmentation. Nat. Methods.

[40] He K., Gkioxari G., Dollar P., Girshick R. (2020). Mask R-CNN. IEEE Trans Pattern
Anal Mach Intell.

[41] Badrinarayanan V., Kendall A., Cipolla R. (2017). SegNet: A Deep Convolutional
Encoder-Decoder
Architecture for Image Segmentation. IEEE Trans
Pattern Anal Mach Intell.

[42] Chen L. C., Papandreou G., Kokkinos I., Murphy K., Yuille A. L. (2018). DeepLab:
Semantic Image Segmentation with Deep Convolutional Nets, Atrous Convolution,
and Fully Connected CRFs. IEEE Trans Pattern
Anal Mach Intell.

[43] Rajchl M., Lee M. C., Oktay O., Kamnitsas K., Passerat-Palmbach J., Bai W., Damodaram M., Rutherford M. A., Hajnal J. V., Kainz B. (2017). DeepCut:
Object Segmentation From Bounding Box Annotations Using Convolutional
Neural Networks. IEEE Trans Med. Imaging.

[44] Archit A., Freckmann L., Nair S., Khalid N., Hilt P., Rajashekar V., Freitag M., Teuber C., Spitzner M., Tapia Contreras C. (2025). Segment Anything for Microscopy. Nat. Methods.

[45] Pachitariu M., Rariden M., Stringer C. (2025). Cellpose-SAM:
superhuman generalization
for cellular segmentation. bioRxiv.

[46] Bilodeau A., Beaupré F., Chabbert J., Thériault K., Deschênes A., Bellavance J.-M., Lessard K., Bernatchez R., De Koninck P., Gagné C. (2026). A Self-Supervised Foundation
Model for Robust and Generalizable Representation Learning in STED
Microscopy. bioRxiv.

[47] Aflalo A., Kashti T., Eldar Y. (2023). DeepCut: Unsupervised
Segmentation
using Graph Neural Networks Clustering. ArXiv.

[48] Sommer C., Hoefler R., Samwer M., Gerlich D. W. (2017). A deep learning
and novelty detection framework for rapid phenotyping in high-content
screening. Mol. Biol. Cell.

[49] Liu C. C., Greenwald N. F., Kong A., McCaffrey E. F., Leow K. X., Mrdjen D., Cannon B. J., Rumberger J. L., Varra S. R., Angelo M. (2023). Robust phenotyping of highly multiplexed
tissue imaging data using pixel-level clustering. Nat. Commun..

[50] Guan S., Knapp T., Alfonso-Garcia A., Duan S., Sawyer T. W. (2025). Optical
Phenotyping Using Label-Free Microscopy and Deep Learning. bioRxiv.

[51] Zhang S., Coffin C., Rogers K. L., Royer C. A., Wang G. (2026). Artificial-intelligence-driven
segmentation and analysis of microbial cells. J. Biomed. Opt..

[52] Park S., Gach H. M., Kim S., Lee S. J., Motai Y. (2021). Autoencoder-Inspired
Convolutional Network-Based Super-Resolution Method in MRI. IEEE J. Transl Eng. Health Med..

[53] Feng F., Liang S., Luo J., Chen S. L. (2022). High-fidelity
deconvolution
for acoustic-resolution photoacoustic microscopy enabled by convolutional
neural networks. Photoacoustics.

[54] Zhang L., Dai H., Sang Y. (2022). Med-SRNet: GAN-Based
Medical Image Super-Resolution
via High-Resolution Representation Learning. Comput. Intell Neurosci.

[55] Zhong E. D., Bepler T., Berger B., Davis J. H. (2021). CryoDRGN: reconstruction
of heterogeneous cryo-EM structures using neural networks. Nat. Methods.

[56] Levy A., Raghu R., Feathers J. R., Grzadkowski M., Poitevin F., Johnston J. D., Vallese F., Clarke O. B., Wetzstein G., Zhong E. D. (2025). CryoDRGN-AI: neural ab initio reconstruction
of challenging cryo-EM and cryo-ET datasets. Nat. Methods.

[57] Sanchez-Garcia R., Gomez-Blanco J., Cuervo A., Carazo J. M., Sorzano C. O. S., Vargas J. (2021). DeepEMhancer: a deep learning solution for cryo-EM
volume post-processing. Commun. Biol..

[58] Webber G., Mizuno Y., Howes O. D., Hammers A., King A. P., Reader A. J. (2025). Likelihood-Scheduled
Score-Based Generative Modeling
for Fully 3D PET Image Reconstruction. IEEE
Transactions on Medical Imaging.

[59] Morgado L., Gomez-de-Mariscal E., Heil H. S., Henriques R. (2024). The rise of
data-driven microscopy powered by machine learning. J. Microsc.

[60] Danial J.
S. H. (2025). Super-resolution
microscopy for structural biology. Nat. Methods.

[61] Sigal Y. M., Zhou R., Zhuang X. (2018). Visualizing
and discovering cellular
structures with super-resolution microscopy. Science.

[62] Liu T., Liu J., Li D., Tan S. (2025). Bayesian deep-learning structured
illumination microscopy enables reliable super-resolution imaging
with uncertainty quantification. Nat. Commun..

[63] Ebrahimi V., Stephan T., Kim J., Carravilla P., Eggeling C., Jakobs S., Han K. Y. (2023). Deep learning
enables
fast, gentle STED microscopy. Commun. Biol..

[64] Heine J., Reuss M., Harke B., D’Este E., Sahl S. J., Hell S. W. (2017). Adaptive-illumination
STED nanoscopy. Proc. Natl. Acad. Sci. U. S.
A..

[65] Hümpfer N., Thielhorn R., Ewers H. (2024). Expanding boundaries - a cell biologist’s
guide to expansion microscopy. J. Cell Sci..

[66] Chowdhury R., Krah D., Ntolkeras A., Heimbrodt A., Shaib A. H. (2025). High Precision Antibody-Free Microtubule Labeling for
Expansion Microscopy. Bio-Protoc..

[67] Chowdhury R., Mimoso T., Chouaib A. A., Mougios N., Krah D., Opazo F., Köster S., Rizzoli S. O., Shaib A. H. (2025). Microtubules
as a versatile reference standard for expansion microscopy. Commun. Biol..

[68] Shaib A. H., Chouaib A. A., Chowdhury R., Altendorf J., Mihaylov D., Zhang C., Krah D., Imani V., Spencer R. K. W., Georgiev S. V. (2025). One-step
nanoscale expansion
microscopy reveals individual protein shapes. Nat. Biotechnol..

[69] Vaswani A., Shazeer N., Parmar N., Uszkoreit J., Jones L., Gomez A. N., Kaiser L., Polosukhin I. (2017). Attention
Is All You Need. ArXiv.

[70] Dosovitskiy A., Beyer L., Kolesnikov A., Weissenborn D., Zhai X., Unterthiner T., Dehghani M., Minderer M., Heigold G., Gelly S. (2020). An Image is Worth 16
× 16 Words: Transformers for Image Recognition at Scale. ArXiv.

[71] Sekh A. A., Opstad I. S., Godtliebsen G., Birgisdottir Å. B., Ahluwalia B. S., Agarwal K., Prasad D. K. (2021). Physics-based
machine
learning for subcellular segmentation in living cells. Nature Machine Intelligence.

[72] Ding Y., Li J., Zhang J., Li P., Bai H., Fang B., Fang H., Huang K., Wang G., Nowell C. J. (2025). Mitochondrial segmentation and function prediction
in live-cell images
with deep learning. Nat. Commun..

[73] Garcia S. B., Schlotter A. P., Pereira D., Recupero A. J., Polleux F., Hammond L. A. (2025). RESPAN: A Deep Learning Pipeline
for Accurate and Automated
Restoration, Segmentation, and Quantification of Dendritic Spines. bioRxiv.

[74] Shen B., Liu S., Li Y., Pan Y., Lu Y., Hu R., Qu J., Liu L. (2022). Deep learning
autofluorescence-harmonic microscopy. Light
Sci. Appl..

[75] Gyawali R., Dhakal A., Wang L., Cheng J. (2024). CryoSegNet: accurate
cryo-EM protein particle picking by integrating the foundational AI
image segmentation model and attention-gated U-Net. Brief Bioinform.

[76] Cameron C. J. F., Seager S. J. H., Sigworth F. J., Tagare H. D., Gerstein M. B. (2024). REliable
PIcking by Consensus (REPIC): a consensus methodology for harnessing
multiple cryo-EM particle pickers. Commun. Biol..

[77] Buchholz T. O., Krull A., Shahidi R., Pigino G., Jekely G., Jug F. (2019). Content-aware image
restoration for electron microscopy. Methods
Cell Biol..

[78] Saal K. A., Shaib A. H., Mougios N., Crzan D., Opazo F., Rizzoli S. O. (2023). Heat denaturation
enables multicolor X10-STED microscopy. Sci.
Rep..

[79] Chouaib A. A., Chang H.-F., Khamis O. M., Alawar N., Echeverry S., Demeersseman L., Elizarova S., Daniel J. A., Tian Q., Lipp P. (2025). Highly adaptable deep-learning platform for automated
detection and analysis of vesicle exocytosis. Nat. Commun..

[80] Jiang X., Li Y., Tian X., Yang S., Luo R., Zhou C., Liu Y., Hu J., Feng S., Gan L. (2026). AI-Driven
Acceleration of Fluorescence Probe Discovery. Adv. Sci..

[81] Jonathan
Ho A. J. (2020). Pieter Abbeel. Denoising Diffusion Probabilistic Models. ArXiv.

[82] Fan H., Xu P., Chen X., Li Y., Zhang Z., Hsu J., Le M., Ye E., Gao B., Demos H. (2023). Mask R-CNN
provides efficient and accurate measurement of chondrocyte viability
in the label-free assessment of articular cartilage. Osteoarthr. Cartil. Open.

[151] Fahmid Hossain, F. M. ; Md Imtiaz, S. ; Lee, K.-Y. ; Wu, H.-Y. ; Kwon, K.-C. ; Kim, N. U^2^-Net Architecture Contingent Intelligent Depth Map Extraction Method Using Light Field Images. In 2024 Conference on Lasers and Electro-Optics Pacific Rim (CLEO-PR); IEEE, 2024. https://ieeexplore.ieee.org/document/10676633 (accessed 2026-06-30).

[83] Wei-Lin
Chiang X. L., Si Si, Li Y., Bengio S., Hsieh C.-J. (2019). Cluster-GCN: An Efficient Algorithm for Training Deep
and Large Graph Convolutional Networks. ArXiv.

[84] Zhu Y., Fang J., Ahmed S. A. H., Zhang T., Zeng S., Liao J.-Y., Ma Z., Qian L. (2025). A modular artificial
intelligence framework to facilitate fluorophore design. Nat. Commun..

[85] Scott
Lundberg S.-I. L. (2017). A Unified Approach to Interpreting Model Predictions. ArXiv.

[86] Hu Q., Hailstone M., Wang J., Wincott M., Stoychev D., Atilgan H., Gala D., Chaiamarit T., Parton R. M., Antonello J. (2023). Universal adaptive optics
for microscopy through embedded neural network control. Light Sci. Appl..

[87] Rates A., Passmore J. B., Norlin N., Kapitein L. C. (2026). Smart microscopy:
adaptive microscope control to improve the way we see life. Npj Imaging.

[88] Jun J. V., Chenoweth D. M., Petersson E. J. (2020). Rational design of small molecule
fluorescent probes for biological applications. Org. Biomol. Chem..

[89] Jiang G., Liu H., Liu H., Ke G., Ren T. B., Xiong B., Zhang X. B., Yuan L. (2024). Chemical Approaches to Optimize the
Properties of Organic Fluorophores for Imaging and Sensing. Angew. Chem. Int. Ed..

[90] Huang S., Huang W., Fang Y., Zhu Y., Huang J., Chen F., Dong J., Zeng W. (2025). Interpretable Artificial
Intelligence Decodes the Chemical Structural Essence of Twisted Intramolecular
Charge Transfer and Planar Intramolecular Charge Transfer Fluorophores. Research.

[91] Lu Y., Huang W., Li Y., Xu Y., Wei Q., Sha C., Guo P. (2025). Leveraging artificial
intelligence in antibody-drug
conjugate development: from target identification to clinical translation
in oncology. npj Precis. Oncol..

[92] Croitoru A., Orr A. A., MacKerell A. D. (2025). Harnessing
computational technologies
to facilitate antibody-drug conjugate development. Nat. Chem. Biol..

[93] Romero P. A., Arnold F. H. (2009). Exploring protein fitness landscapes
by directed evolution. Nat. Rev. Mol. Cell Biol..

[94] Butcher J., Krishna R., Mitra R., Brent R. I., Li Y., Corley N., Kim P. T., Funk J., Mathis S., Salike S. (2025). De novo
Design of All-atom Biomolecular Interactions
with RFdiffusion3. bioRxiv.

[95] Watson J. L., Juergens D., Bennett N. R., Trippe B. L., Yim J., Eisenach H. E., Ahern W., Borst A. J., Ragotte R. J., Milles L. F. (2023). De
novo design of protein structure and function
with RFdiffusion. Nature.

[96] Wei
Qu Y. M., Ye F., Lu C., Zhou Yi, Zhang K., Wang L., Gui M., Gu Q. (2026). SeedProteo:
Accurate De Novo All-Atom Design of Protein Binders. ArXiv.

[97] Sappington I., Toul M., Lee D. S., Robinson S. A., Goreshnik I., McCurdy C., Chan T. C., Buchholz N., Huang B., Vafeados D. (2026). Improved
protein binder design using β-pairing
targeted RFdiffusion. Nat. Commun..

[98] Lin Z., Akin H., Rao R., Hie B., Zhu Z., Lu W., Smetanin N., Verkuil R., Kabeli O., Shmueli Y. (2023). Evolutionary-scale prediction of atomic-level protein structure with
a language model. Science.

[99] Swanson D. R. (1990). Medical
literature as a potential source of new knowledge. Bull. Med. Libr. Assoc..

[100] De Sa C., Ratner A., Re C., Shin J., Wang F., Wu S., Zhang C. (2016). DeepDive: Declarative
Knowledge Base Construction. SIGMOD Rec.

[101] Ammar, W. ; Groeneveld, D. ; Bhagavatula, C. ; Beltagy, I. ; Crawford, M. ; Downey, D. ; Dunkelberger, J. ; Elgohary, A. ; Feldman, S. ; Ha, V. ; Construction of the Literature Graph in Semantic Scholar. In New Orleans - Louisiana; Association for Computational Linguistics, 2018; pp 84–91.

[102] Yeganova L., Kim W., Tian S., Comeau D. C., Wilbur W. J., Lu Z. (2025). LitSense 2.0: AI-powered
biomedical
information retrieval with sentence and passage level knowledge discovery. Nucleic Acids Res..

[103] Freeman J., Millikin R. J., Xu L., Sharma I., Moore B., Lock C., Shine George K., Bal A., Mohanty C., Stewart R. (2026). SKiM-GPT: combining biomedical literature-based
discovery with large language model hypothesis evaluation. BMC Bioinf..

[104] Xu Q., Soto C., Shahnawaz M., Liu X., Jiang X., Kim Y. (2025). Multi agent large language models
for biomedical hypothesis generation
in drug combination discovery. iScience.

[105] Kobak D., Gonzalez-Marquez R., Horvat E. A., Lause J. (2025). Delving into
LLM-assisted writing in biomedical publications through excess vocabulary. Sci. Adv..

[106] Lee P. Y., Salim H., Abdullah A., Teo C. H. (2023). Use of
ChatGPT in medical research and scientific writing. Malays. Fam. Physician.

[107] Misra D. P., Chandwar K. (2023). ChatGPT, artificial
intelligence
and scientific writing: What authors, peer reviewers and editors should
know. J. R Coll Physicians Edinb.

[108] Ovelman C., Kugley S., Gartlehner G., Viswanathan M. (2024). The use of a large language model to create plain language
summaries of evidence reviews in healthcare: A feasibility study. Cochrane Evid Synth Methods.

[109] Hwang T., Aggarwal N., Khan P. Z., Roberts T., Mahmood A., Griffiths M. M., Parsons N., Khan S. (2024). Can ChatGPT
assist authors with abstract writing in medical journals? Evaluating
the quality of scientific abstracts generated by ChatGPT and original
abstracts. PLoS One.

[110] Barrington N. M., Gupta N., Musmar B., Doyle D., Panico N., Godbole N., Reardon T., D’Amico R. S. (2023). A Bibliometric
Analysis of the Rise of ChatGPT in Medical Research. Med. Sci..

[111] Jiang Q., Karniadakis G. (2025). AgenticSciML:
Collaborative Multi-Agent
Systems for Emergent Discovery in Scientific Machine Learning. ArXiv.

[112] Hao Li H. C., Peng S., Liu Z., Feng B., Wang Yu, Yan Z., Tian Y., Li Yu, Yuan Li (2026). Agentic reinforcement learning empowers next-generation
chemical
language models for molecular design and synthesis. ArXiv.

[113] Du Y. (2026). Accelerating Scientific
Discovery with Autonomous Goal-evolving
Agents. ArXiv.

[114] Ali Essam Ghareeb B. C., Mitchener L., Yiu A., Szostkiewicz C. J., Laurent J. M., Razzak M. T., White A. D., Hinks M. M., Rodriques S. G. (2025). Robin: A multi-agent system for automating scientific
discovery. ArXiv.

[115] Boiko D. A., MacKnight R., Kline B., Gomes G. (2023). Autonomous
chemical research with large language models. Nature.

[116] M. Bran A., Cox S., Schilter O., Baldassari C., White A. D., Schwaller P. (2024). Augmenting
large language models
with chemistry tools. Nature Machine Intelligence.

[117] Szymanski N. J., Rendy B., Fei Y., Kumar R. E., He T., Milsted D., McDermott M. J., Gallant M., Cubuk E. D., Merchant A. (2023). An autonomous
laboratory for the accelerated
synthesis of inorganic materials. Nature.

[118] Romera-Paredes B., Barekatain M., Novikov A., Balog M., Kumar M. P., Dupont E., Ruiz F. J. R., Ellenberg J. S., Wang P., Fawzi O. (2024). Mathematical discoveries
from program search with large language models. Nature.

[119] Novikov A., Vũ N., Eisenberger M., Dupont E., Huang P.-S., Wagner A. Z., Shirobokov S., Kozlovskii B., Ruiz F. J. R., Mehrabian A. (2025). AlphaEvolve: A coding agent for scientific and algorithmic discovery. ArXiv.

[120] O’Neill C. (2025). Sparks of Science: Hypothesis
Generation Using
Structured Paper Data. ArXiv.

[121] Fromer J. C., Coley C. W. (2023). Computer-aided multi-objective optimization
in small molecule discovery. Patterns.

[122] Li S. (2025). SciLitLLM: How to Adapt
LLMs for Scientific Literature
Understanding. ArXiv.

[123] Liu H. (2025). Literature Meets Data: A Synergistic Approach to Hypothesis
Generation. ArXiv.

[124] Li Y., Gu T., Yang C., Li M., Wang C., Yao L., Gu W., Sun D. (2025). AI-Assisted Hypothesis Generation
to Address Challenges in Cardiotoxicity Research: Simulation Study
Using ChatGPT With GPT-4o. J. Med. Internet
Res..

[125] Bepler T., Morin A., Rapp M., Brasch J., Shapiro L., Noble A. J., Berger B. (2019). Positive-unlabeled
convolutional neural networks for particle picking in cryo-electron
micrographs. Nat. Methods.

[126] Pfab J., Phan N. M., Si D. (2021). DeepTracer
for fast
de novo cryo-EM protein structure modeling and special studies on
CoV-related complexes. Proc. Natl. Acad. Sci.
U. S. A..

[127] Jumper J., Evans R., Pritzel A., Green T., Figurnov M., Ronneberger O., Tunyasuvunakool K., Bates R., Žídek A., Potapenko A. (2021). Highly accurate protein structure prediction
with AlphaFold. Nature.

[128] Abramson J., Adler J., Dunger J., Evans R., Green T., Pritzel A., Ronneberger O., Willmore L., Ballard A. J., Bambrick J. (2024). Accurate
structure prediction of biomolecular interactions with AlphaFold 3. Nature.

[129] Corum M. R. (2024). Predictive modeling and cryo-EM: A synergistic
approach to modeling macromolecular structure. Biophys. J..

[130] Zhang C., Condon A., Dao Duc K. (2025). A comprehensive
survey
and benchmark of deep learning-based methods for atomic model building
from cryo-electron microscopy density maps. Brief Bioinform.

[131] Januszewski M., Li P., Kornfeld Jörgen, Denk W., Jain V. (2016). Flood-Filling Networks. ArXiv.

[132] Shah U., Alzubaidi M., Agus M., Calí C., Magistretti P. J., Househ M. (2025). Deep learning for brain electron
microscopy segmentation: Advances, challenges, and future directions
in connectomics and ultrastructure analysis. Comput. Graph..

[133] Vogt N. (2018). The whole fly brain in detail. Nat. Methods.

[134] Bae J. A., Baptiste M., Baptiste M. R., Bishop C. A., Bodor A. L., Brittain D., Brooks V., Buchanan J., Bumbarger D. J., Castro M. A. (2025). Functional
connectomics
spanning multiple areas of mouse visual cortex. Nature.

[135] Park S. Y., Sheridan A., An B., Jarvis E., Lyudchik J., Patton W., Axup J. Y., Chan S. W., Damstra H. G. J., Leible D. (2025). Combinatorial
protein
barcodes enable self-correcting neuron tracing with nanoscale molecular
context. bioRxiv.

[136] Lee S., Roh S., Woo H., Lee G., Jung H. G., Lee K.-B., Lee H., Yoon D. S., Lee J. H. (2026). AI in Atomic
Force Microscopy: Advancing Biological Nanoscale Imaging and Autonomous
Discovery. ACS Nano.

[137] Zhou Y., Lai M. (2024). Study on Scanning Probe-Induced Electrochemical
Deposition of Atomic and Close-to-Atomic-Scale Structures. Nanomanuf. Metrol..

[138] Wei Z., Wei S., Zeng Q., Lu W., Liu H., Zeng K. (2025). A Novel Deep Reinforcement Learning
Approach for Dynamic Proportional-Integral
Control in Scanning Probe Microscopy. Small.

[139] Natinsky E., Khan R. M., Wang Q., Morris D., Cullinan M., Dingreville R. (2026). AFM-net: machine
learning acceleration
of atomic force microscopy nanometrology from scarce data and fast
scans. npj Comput. Mater..

[140] Jung H., Han G., Jung S. J., Han S. W. (2022). Comparative
study of deep learning algorithms for atomic force microscopy image
denoising. Micron.

[141] Sokolov I. (2024). On machine
learning analysis of atomic force microscopy
images for image classification, sample surface recognition. Phys. Chem. Chem. Phys..

[142] He X., Meile C., Bhandarkar S. M. (2022). Multimodal registration of FISH and
nanoSIMS images using convolutional neural network models. ArXiv.

[143] Masud N., Rade J., Hasib M. H. H., Krishnamurthy A., Sarkar A. (2024). Machine learning approaches
for improving atomic force
microscopy instrumentation and data analytics. Front. Phys..

[144] van Gerwen P., Briling K. R., Bunne C., Somnath V. R., Laplaza R., Krause A., Corminboeuf C. (2024). 3DReact: Geometric
Deep Learning for Chemical Reactions. J. Chem.
Inf. Model..

[145] Amirian M., Barco D., Herzig I., Schilling F.-P. (2024). Artifact
Reduction in 3D and 4D Cone-Beam Computed Tomography Images With Deep
Learning: A Review. IEEE Access.

[146] Webber G., Mizuno Y., Howes O. D., Hammers A., King A. P., Reader A. J. (2025). Likelihood-Scheduled
Score-Based
Generative Modeling for Fully 3D PET Image Reconstruction. IEEE Trans Med. Imaging.

[147] Song Y., Ermon S. (2019). Generative Modeling by Estimating
Gradients of the Data Distribution. ArXiv.

[148] Altabella L., Benetti G., Camera L., Cardano G., Montemezzi S., Cavedon C. (2022). Machine learning for multi-parametric
breast MRI: radiomics-based approaches for lesion classification. Phys. Med. Biol..

[149] Sheth D., Giger M. L. (2020). Artificial intelligence in the interpretation
of breast cancer on MRI. J. Magn. Reson. Imaging.

[150] Al-Karawi D., Al-Zaidi S., Helael K. A., Obeidat N., Mouhsen A. M., Ajam T., Alshalabi B. A., Salman M., Ahmed M. H. (2024). A Review of Artificial Intelligence
in Breast Imaging. Tomography.

